# Proof of concept and early development stage of market-oriented high iron and zinc rice expressing dicot ferritin and rice nicotianamine synthase genes

**DOI:** 10.1038/s41598-022-26854-z

**Published:** 2023-01-12

**Authors:** Nikolaos Tsakirpaloglou, Gela Myan Bueno-Mota, Jessica Candace Soriano, Erwin Arcillas, Felichi Mae Arines, Su-May Yu, James Stangoulis, Kurniawan Rudi Trijatmiko, Russell Reinke, Joseph Tohme, Howarth Bouis, Inez H. Slamet-Loedin

**Affiliations:** 1 International Rice Research Institute (IRRI), Metro Manila, The Philippines; 2 Institute of Molecular Biology, Academia Sinica, Naknag, Taipei, Taiwan, ROC; 3 College of Science and Engineering, Flinders University, Bedford Park, SA Australia; 4 Bioversity International and International Center for Tropical Agriculture (CIAT) Alliance, Cali, Colombia; 5 International Food Policy Research Institute (IFPRI) - Emeritus Fellow, Washington, DC USA; 6 Present Address: Crop Genome Editing Laboratory (CGEL), Soil and Crop Sciences Department, Texas A&M University and Texas A&M AgriLife Research, College Station, TX USA; 7 Present Address: Board Institute of MIT and Harvard, Cambridge, MA USA

**Keywords:** Plant biotechnology, Agricultural genetics, Field trials, Molecular engineering in plants

## Abstract

Micronutrient deficiencies such as iron (Fe), zinc (Zn), and vitamin A, constitute a severe global public health phenomenon. Over half of preschool children and two-thirds of nonpregnant women of reproductive age worldwide have micronutrient deficiencies. Biofortification is a cost-effective strategy that comprises a meaningful and sustainable means of addressing this issue by delivering micronutrients through staple foods to populations with limited access to diverse diets and other nutritional interventions. Here, we report on the proof-of-concept and early development stage of a collection of biofortified rice events with a high density of Fe and Zn in polished grains that have been pursued further to advance development for product release. In total, eight constructs were developed specifically expressing dicot ferritins and the rice nicotianamine synthase 2 (*OsNAS2*) gene under different combinations of promoters. A large-scale transformation of these constructs to Bangladesh and Philippines commercial *indica* cultivars and subsequent molecular screening and confined field evaluations resulted in the identification of a pool of ten events with Fe and Zn concentrations in polished grains of up to 11 μg g^−1^ and up to 37 μg g^−1^, respectively. The latter has the potential to reduce the prevalence of inadequate Zn intake for women of childbearing age in Bangladesh and in the Philippines by 30% and 50%, respectively, compared to the current prevalence. To our knowledge, this is the first potential biotechnology public-sector product that adopts the product cycle phase-gated approach, routinely applied in the private sector.

## Introduction

Micronutrient malnutrition or deficiencies in key micronutrients such as Fe, Zn, and vitamin A is estimated to affect approximately 1.5 billion people globally, particularly in developing countries^1^. The term is synonymous with “hidden hunger” which refers to the invisible nature of this condition and the lack of obvious and evident symptoms^2,3^. Pregnant women and children are the most vulnerable groups of the population and the ones who suffer the greatest adverse effects^4^. Recently published findings revealed that 372 million preschool-aged children and 1.2 billion non-pregnant women of reproductive age are affected worldwide by at least one of the three micronutrient deficiencies^1^. The impact at a country level is associated with economic losses in the range of 2 to 5% of their gross domestic product (GDP)^5^.

Fe deficiency is the major cause of anemia and indiscriminately affects approximately 1 billion people worldwide, at all life stages, and across gender^1,6,7^. The estimates of WHO are staggering particularly for young children and pregnant women, since 42% of children less than 5 years of age and 40% of pregnant women worldwide are anemic^8^. These observations become more somber in developing countries like Bangladesh^9^ or in the Philippines^10^.

Zn deficiency is a major cause of stunting in children^11,12^. A joint effort from UNICEF, WHO, and the World Bank released recently estimated that stunting affects 144 million children globally (or 21.3%) under the age of 5 years old^13^. The prevalence of Zn deficiency in Bangladesh is remarkably high since it affects approximately half of the children and pregnant women^9^. In the Philippines, the numbers are still alarming, despite a reduction in the prevalence of stunting from 33.4% in 2015 to 30.3% in 2018 among children under five years old^10^.

Addressing micronutrient malnutrition is a crucial element for achieving the Sustainable Development Goals (SDGs) by 2035^14^. Several strategies have been employed to tackle micronutrient deficiencies including supplementation as well as food-based approaches such as dietary diversification, food fortification, and biofortification^15^. Biofortification, the process of breeding nutrients into staple food crops, is a feasible and cost-effective means of delivering micronutrients mainly to rural populations of developing countries with limited access to other micronutrient interventions^16^. In combination with the existing interventions, biofortification has the potential in reducing the burden of maternal and child morbidity, impaired neurocognitive development, and mortality^17–19^. Since 2003, HarvestPlus and its partners have demonstrated the potential of this agriculture-based method for addressing micronutrient deficiency through plant breeding.

Conventional breeding efforts for developing Fe-enriched varieties were hampered because of the limited variability in Fe concentration in polished grains among rice germplasms^20^. Recently, high-Zn rice varieties were released under the HarvestPlus program for commercial cultivation in Bangladesh, India, and the Philippines^21^. Although these varieties have an increased content of Zn (up to 23 µg g^−1^ in the Philippines (personal communication with BP Mallikarjuna Swamy, IRRI) and up to 27.6 µg g^−1^ in Bangladesh^22^) compared to the baseline detected in popular varieties, they are borderline reaching, in the case of Bangladesh, or are still slightly below the dietary target of 28 μg g^−1^ that is required to achieve the 30% of estimated average requirement (EAR) for non-pregnant, non-lactating women and children 4–6 years of age and to subsequently maximize the biological impact of this intervention^23^. Utilization of genetic engineering advances to improve the Fe content of rice was initiated at the beginning of our century^24^ and throughout this period several approaches were tested and considered^25–27^. The loading of Fe and Zn in developing rice seeds occurs mainly through the phloem pathway through attachment to metal chelators such as nicotianimine (NA)^28,29^. Recently, we reported on the development of a biofortified *indica* rice that attains the nutritional Fe targets under confined field settings^30^. The presence of *OsNAS2* gene, the precursor of NA, also resulted in the concomitant increase of Zn levels in polished rice grains. Most importantly, this increase in Fe and Zn concentrations in rice seeds was shown to be bioavailable^30^ and to be retained after cooking^21^.

The cost and complexity of translating basic science advances to the agricultural biotechnology sector are often underestimated, resulting in rare examples of applications. The R&D pipeline for agriculture biotech products is broadly characterized by three stages, Research, Development, and Commercialization^31,32^. Advancement of a potential product through this pipeline involves the fulfillment of certain prerequisites including thorough testing, the satisfaction of product concept requirements, and assurance for safety^31,33^. For this purpose, a large number of events are often required to be developed for testing to apply rigorous selection to obtain candidates suitable for commercialization and adoption^34^. This might be an additional reason for hindering the involvement of public sector in a genetically modified (GM) crop pipeline^35^.

We aim to develop a low-cost inbred biofortified (Fe-/Zn-dense) rice in popular *indica* varieties by translating the advances of fundamental science into a potential product, suitable for release. The product concept consists of achieving the Fe and Zn dietary targets of 10 μg g^−1^ for Fe (from the baseline of 2 μg g^−1^) and 28 μg g^−1^ for Zn (from the baseline of 12 μg g^−1^)^23^, through a well-defined single-locus transgene integration, and a good and stable agronomic performance under confined field settings. Earlier, we were able to fulfill the prerequisites of "proof-of-concept”^30^. Nonetheless, soybean is considered one of the top 10 allergen sources according to the US Food Allergen Labelling and Consumer Protection Act of 2004 (FALCPA)^36^ and hence utilization of the soybean ferritin gene could potentially extend the approval process for the development and acceptance of a potential product. We were, therefore, compelled to explore and consider alternative ferritin protein sources that would ease the commercialization, and adoption of the potential product. At the same time, we decided to diversify the promoters used to drive the transgenes.

**Figure 1:**
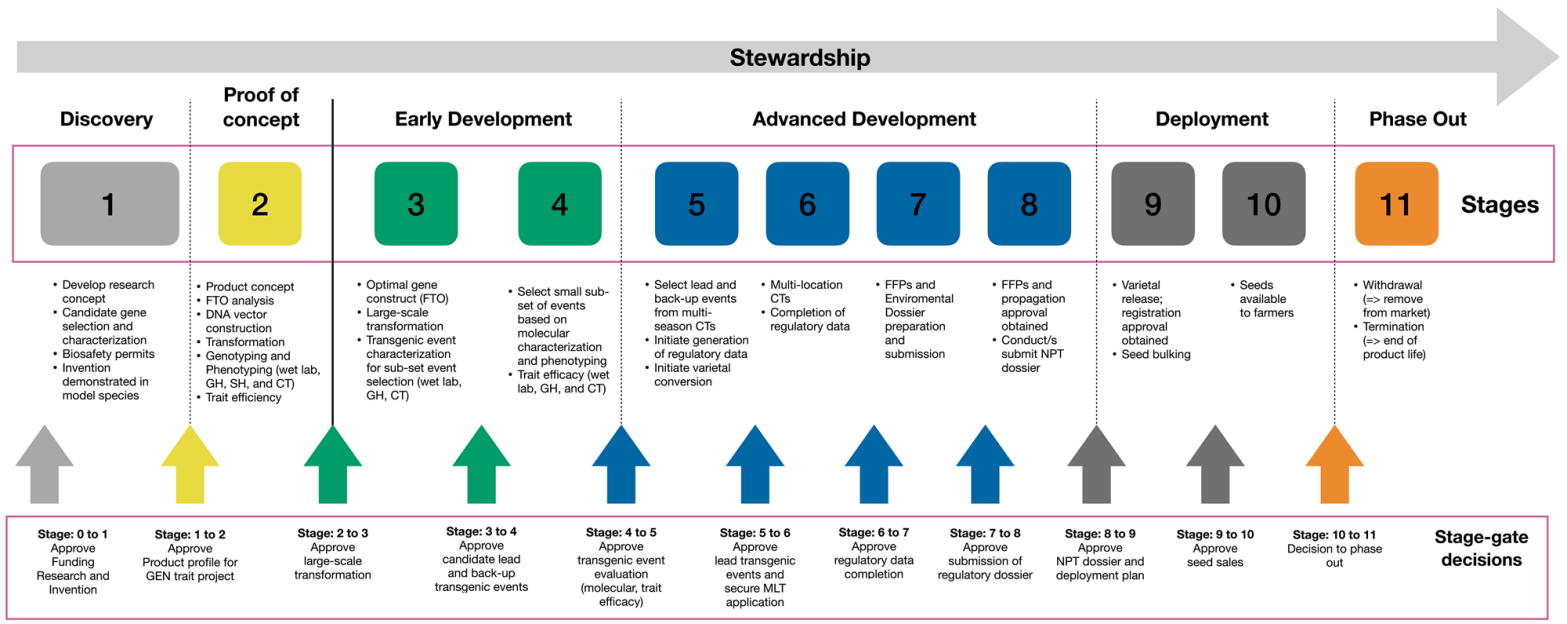
The life cycle of a transgenic product development. A stepwise process that involves several stages for transgenic product development. Advancement from one stage to the next is determined by thorough data analysis and fulfillment of certain criteria, specific for each stage. CT: Confined test on the field; FFP: food, feed, and processing; FTO: freedom to operate; GEN: Genetic engineering; GH; greenhouse; MLT: multi-location CT; NPT: national performance trial; SH: screenhouse.

Here, we report on the proof-of-concept and the early development stage of potential candidate Fe- and Zn-dense biofortified events with novel combinations of constitutive and phloem-specific promoter sequences regulating the expression of *OsNAS2* gene, as well as the utilization of ferritin gene orthologues from commonly consumed dicot species, suitable for future release. For this purpose, we adopted a phase-gated process (Fig. 1), a regular practice for transgenic product development in private sector, but not in the public sector, to track the life-cycle development of the product. Moreover, for broader and immediate impact, we introduced the cassettes directly to widely grown varieties for Bangladesh (cv. BRRI Dhan28) and the Philippines (cv. NSIC Rc238). This is a step closer for Fe- and Zn-dense rice to reach the rural areas in these countries and to contribute to improving the life quality, whilst reducing the economic losses associated with micronutrient deficiency^37^.

## Materials and methods

All the methods reported below were performed according to the relevant guidelines and regulations.

### Similarity and allergenicity assessment of selected dicot ferritin proteins

The amino acid sequence of the SFerH-1 protein was analyzed using the Basic Local Alignment Search Tool (BLAST) of the UniProt database (www.uniprot.org/) to identify similar, previously reviewed, ferritin proteins from other crop species. To assess the potential allergenic cross-reactivity of the selected dicot ferritin proteins their amino acid sequences were compared to a peer-reviewed database of 2171 known and putative allergen and celiac protein sequences residing in the FARRP20 dataset at the University of Nebraska-Lincoln^38^. Potential identities between the selected ferritins and proteins in the allergen database were evaluated with the FASTA35 sequence alignment tool using the default parameters^39^. The recommended greater than 35% identity threshold over any 80-amino acid length sequence alignment between the query sequence and an allergen was used to indicate the potential for cross-reactivity.

### Generation of plant transformation vectors and transgenic rice

**Figure 2:**
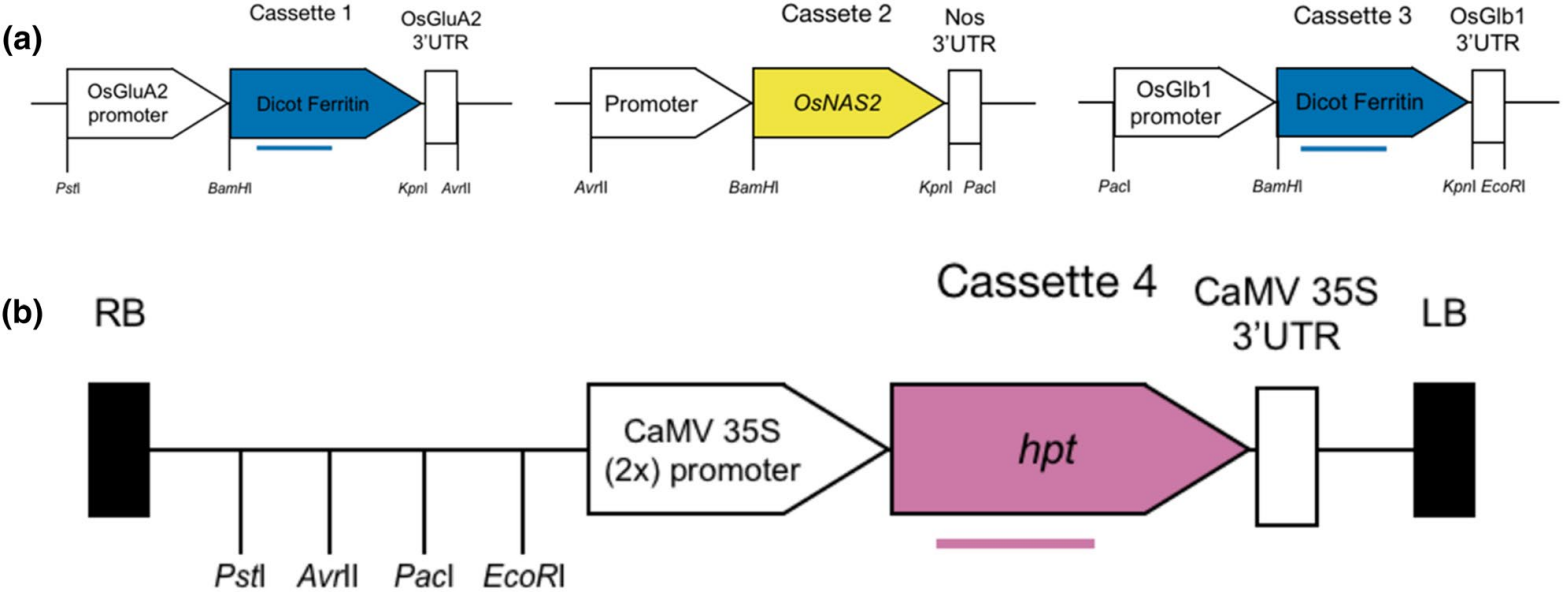
Schematic diagram of the T-DNA constructs prepared for rice transformation. (**a**) The dicot ferritin was either *ApFer*, *PvFer* or *PsFer*, whilst the promoter in cassette 2 is either the phloem specific OsTRXh promoter in combination or not with the maize ubiquitin intron, or the constitutive CaMV 35S promoter (single enhancer). The cassettes 1, 2 and 3 were initially assembled and cloned in TOPO vectors with the indicated overhangs. (**b**) The cassettes were introduced to the pCambia-based backbone by directional cloning after a series of digestions with respective enzymes and subsequent ligation reactions. The structure and orientation of the whole T-DNA region was confirmed by sequencing prior to rice transformation. RB and LB represent the right and left borders of the T-DNA, respectively; *hpt*: hygromycin phosphotransferase. The horizontal lines below dicot ferritins (blue) or the *hpt* (pink) genes represent the deduced hybridization position of the dioxigenin-labeled cDNA probes.

*Agrobacterium*-mediated transformation was performed using a pCAMBIA1300-based backbone plasmid vector (GenBank Accession No. AF234296), which contained four gene expression cassettes within the T-DNA (Fig. 2). The first cassette contained the chloroplast ferritin (EC 1.16.3.1) encoding gene from *Phaseolus vulgaris* (*PvFer*; Accession No. sp| CAA41213.1), *Malus baccata* var. xiaojinensis (*ApFer*; Accession No. sp| AAK83702.1) or *Pisum sativum* (*PsFer*; Accession No. sp| CAA45763.1) under the control of the rice glutelin (GluA-2) promoter^40^ for targeted expression in the outer portion of rice endosperm. The nucleotide sequence of the *PvFer* gene was identical to the coding sequence of the native gene (GenBank Accession No. X58274.1)^41^, the nucleotide sequence of the *ApFer* gene in IRS1030 was identical to the coding sequence of the native gene (GenBank Accession No. AF315505)^42^, and the nucleotide sequence of the *PsFer* ferritin was identical to the coding sequence of the native gene (GenBank Accession No. X64417.1)^43^. Termination sequences were derived from 3’ untranslated region (3’ UTR) of the rice glutelin (GluA-2) encoding gene^44,45^ to increase the expression of the respective dicot ferritin gene^46^. The structure of the second transgene cassette contained a copy of the nicotianamine synthase 2 (EC 2.5.1.43) encoding gene from *Oryza sativa* (*OsNAS2*) (Accession No. sp|Q10MI9) under the control of the phloem specific Thioredoxin h (OsTRXh) promoter from rice, as well as its 5’ untranslated region^47^, the maize Ubiquitin intron 1 (in IRS1134)^48^ or the cauliflower mosaic virus (CaMV) 35S promoter (single enhancer region). In the latter, transcription termination of the *OsNAS2* gene was provided by the polyadenylation signal and 3’ UTR from the nopaline synthase (nos) gene of *Agrobacterium* *tumefaciens* Ti plasmid pTiT37^49,50^. In the cases of IRS1030, IRS1034, IRS1134, and IRS1202 a synthesized version of the *OsNAS2* gene was used in which the C nucleotide in position 441 of the coding sequence was replaced to T to remove the *Aar*I restriction site, originally present in the native sequence. This change in the coding sequence did not result in any alterations in the amino acid sequence. The third cassette contained another copy of the chloroplastic dicot ferritins (EC 1.16.3.1) encoding the respective genes used in the first cassette under the control of the rice globulin (Glb1) promoter^51^ for targeted expression in the internal portion of rice endosperm. Termination sequences were derived from 3’ UTR of the rice globulin (Glb1) encoding gene^51^ to increase the expression of the respective dicot ferritin genes^46^. The fourth and final cassette contained a copy of the hygromycin phosphotransferase (*hpt*) gene from *Escherichia coli* (GenBank Accession No. Z37515) for positive selection of transformed plants grown on medium containing hygromycin B. Transcription of the *hpt* gene was regulated by the promoter (double enhancer) and polyadenylation regions of the CaMV 35S RNA (GenBank Accession No. AF234296). The dicot ferritin genes, the synthesized version of *OsNAS*2, and the *hpt* gene described earlier were chemically synthesized at Blue Heron Biotech (Bothell, WA, USA) and cloned into pUC vectors with the desirable restriction enzymes. Transformation of *indica* rice cv. BR28 or cv. Rc238 was performed using immature embryos as previously described^52^. As plantlets regenerated and rooted, they were transferred to paddy at the greenhouse. In total, one thousand eight hundred and seventy events from eight constructs were produced and grown in the paddy under greenhouse settings for further analysis. Transgene presence in transformants was confirmed by leaf PCR using KAPA3G Plant PCR Kit (Kapa Biosystems, Catalog number: KK7251) and gene-specific primers for the dicot ferritin for the respective constructs (SferH1-RT_F: 5’-CTTGCTGTTCCAACTGCTCC-3’ and SferH1-RT_R: 5’-ATTGTTGCGATCTGCCACAC-3’ for confirming presence /absence of the *ApFer* and *PsFer* genes, and PvFer_F2: 5’-CCCAACCTGTTCTGTTTCTTTGAGC-3’ and PvFer_R1: 5’-CAACGAAACCTGCCCAGC-3’ for confirming presence/absence of the *PvFer* gene).

### Selection of single-copy events based on qPCR analysis

TaqMan RT-PCR was carried out in a fluorometric thermal cycler (Roche LightCycler 480 system) in 20-µl reaction mixtures containing 1 × TaqMan Genotyping Master Mix (Applied Biosystems, Catalog number: 4371355), 4 µl DNA sample and optimal concentrations of each transgene-specific primer and probe (1 mM each HPTF/HPTR primer and 1 mM HPTP probe^53^ or 1 mM each KVM159/KVM160 primer and 1 mM TM013 probe^54^). The amplification conditions consisted of one cycle of 20 s at 50 °C and 10 min at 95 °C, followed by 40 cycles of 15 s at 95 °C and 1 min at 60 °C. Each sample was quantified in triplicates. The *hpt* copy numbers were calculated using a comparative ΔΔCt method as described previously^55^. Genomic DNA from a known single-copy transgenic plant (the single-copy sample was previously determined by DNA blot analysis) was used as a calibrator.

### Qualitative selection of best constructs and events based on Fe staining

Harvested T_1_ seeds from T_0_ transgenic plants were dehusked to obtain brown seeds. Then, thirty of them were placed in 2-mL Eppendorf tubes which were subsequently transferred in a Cryo-Block for 48 Microcentrifuges (SPEX SamplePrep) and shaken vigorously for 2 min at 1200 strokes per min for 70 cycles using a 2000 Geno/Grinder (Spex SamplePrep). Approximately, ten polished seeds (both broken and unbroken) were then transferred in 6-well plates and incubated in Milli-Q water and the vacuum was applied for 30 min. The Milli-Q water was then removed and a mixture of 4% (w/v) potassium ferrocyanide (K_4_ [Fe (CN)_6_]3H_2_O) with 4% (v/v) HCl (12 N) was added to the polished seeds followed by 30 min vacuum treatment to allow infiltration of the staining solution in the seeds and subsequent incubation in dark at room temperature. Upon development of the blue color, the staining solution was removed, and the polished seeds were washed six times in Milli-Q water before visual observation and scoring under stereoscopes^56^. Polished seeds of event NASFer-274^30^ were used as a positive control in each plate.

### DNA blot analysis

Total genomic DNA was prepared from rice leaves of T_0_ plants as previously described^57^. *Pst*I- or *EcoR*I-digested DNA (10 mg) was separated by agarose electrophoresis, blotted to a nylon membrane, and hybridized by dicot ferritin and a hygromycin probe labeled with dioxigenin (Roche Applied Science, Germany). Hybridization and detection were carried out as previously described^58^. The previously reported gene-specific ferritin primers were used to prepare the gene-specific probes. For the hygromycin (hpt) probe the target region was amplified from plasmid DNA using the NT_hpt_F1 primer (5’-CGCAAGGAATCGGTCAATA-3’) and the NT_hpt_R2 primer (5'-CGTCTGCTGCTCCATACAA-3').

### Quantitative determination of Fe concentration in multiple generations

At IRRI, polished grain samples (0.600–0.625 g) were digested in an ultrapure HNO3-HClO4 mixture in a microwave autoclave (Ultra Clave II, MLS GmbH, Leutkirch, Germany). Fe and Zn concentrations were determined using ICP-OES (Perkin Elmer ICP Optima 5300DV). The National Institute of Standards and Technology (NIST) rice flour standard 1568a and the Wageningen Evaluating Programs for Analytical Laboratories (WEPAL) IPE-135 were used as quality controls, and the samples were digested and analyzed using the same method as used for the rice samples. At Flinders University, ICP-OES of the polished rice grains was performed as it is previously described^59,60^.

### T-DNA flanking sequence recovery

Genomic sequences flanking the T-DNA right and left borders were amplified using TAIL-PCR as described previously^61^. Identification of the insert position in the rice genome was performed using a BlastN algorithm^62^ at the Rice Galaxy resource database^63^.

### Zygosity test of heterozygous plants

Direct leaf PCR using the KAPA3G Plant PCR Kit (KAPA Biosystems) according to manufacturer instructions was used to determine the zygosity of T_1_ and T_2_ plants for selective events. The PCR-based assay included three oligonucleotides per respective event and was performed as previously described^30,34^.

### Seed multiplication and confined field evaluation

Seed multiplication was conducted in a transgenic greenhouse at the Zeigler Experimental Station at IRRI, Philippines. Thirty (30) T_1_ plants per selected event were grown in the paddy and the zygosity of the individual plants was determined by direct leaf PCR, as described earlier. Harvested T_2_ seeds were used for subsequent confined field evaluation. A confined test (CT) on the field was conducted at the Zeigler Experimental Station at IRRI, Philippines (site GPS coordinates: 14° 08′ 56.9″ N and 121° 15′ 16.8″ E; 14°09′ 50.4″ N and 121° 15′ 28.2″ E; 14° 09′ 50.6″ N and 121° 15′ 28.2″ E; 14° 09′ 50.7″ N and 121° 15′ 29.0″ E) between November 2016 and March 2017, using back-crossed homozygous lines of selected events (T_2_). The trials were planted in a randomized complete block design with four replicates. The plot size was 4 rows × 15 hills (25 cm between rows and 20 cm between hills) with one plant per hill. NPK fertilizer (14:14:14) was applied as complete basal fertilizer before leveling at a rate of 30 kg ha^−1^. Two additional splits of urea (46:0:0) were applied after transplanting at a rate of 20 kg ha^−1^ and 40 kg ha^−1^ as a top dressing. Six plants from each plot were randomly chosen for the evaluation of grain yield and other agronomic characteristics. The seeds of the six plants were then bulked and dehulled. The seeds were polished using a modified non-contaminating Kett Mill with a milling time of 2 min 30 s as previously described^30^. Polished grain samples were submitted for metal quantification to IRRI Analytical Service Laboratory (ASL) and the Waite Analytical Service at Flinders University, Australia. In the latter, samples were digested using nitric/perchloric acid on a programmable digestion system in open glass tubes^59^. Fe and Zn concentrations were determined using inductively coupled plasma-atomic emission spectrometry (SPECTRO CIROS Radial).

### Preliminary breeding and trait stability

The selected events were crossed in the paddy under screen house conditions with the recurrent parents cv. BR28 and cv. Rc238, respectively and the identified homozygous BC_2_F_2_ lines were evaluated for agronomic performance and trait stability through field trials at the Zeigler Experimental Station at IRRI, Philippines. The trials were planted in a randomized complete block design with four replicates. The plot size was 4 rows × 15 hills (25 cm between rows and 20 cm between hills) with one plant per hill. Government recommendation of NPKZn fertilizers for rice farmers was followed, and P, K, and Zn were applied at the rate of 150-30-30-5 kg ha^−1^ as basal fertilizer before leveling, whereas N was applied in three splits, once before leveling and twice as a top dressing. Five plants from each plot which were phenotypically similar to the respective wild-type counterparts and were chosen for grain yield evaluation and other agronomic characteristics. The seeds of the five plants were then bulked and dehulled. The seeds were polished using a modified non-contaminating Kett Mill. Polished grain samples were submitted for metal quantification to IRRI ASL.

## Results

### Allergenicity analysis of the apple, pea, and bean ferritin proteins

Allergenicity analysis is a crucial step in the design and development of a transgenic product for market release. Sequence analysis of the SFerH-1 using the Basic Local Alignment Search Tool (BLAST) of the UniProt database revealed identity with 22 previously reviewed Swiss-Prot proteins. In particular, the ApFer (UniProt entry: Q94FY2) exhibited 99.2% identity with the SferH-1, the PvFer (UniProt entry: P25699) was 90.4% identical and the PsFer (UniProt entry: P19975) was 81.2% (Suppl. Table 1). Amino acid sequence alignment of the SferH-1 with the dicot ferritins that exhibited the highest similarity revealed most of the differences in the signal peptide region, whilst the mature peptides remained highly conserved (Suppl. Figure 1).

Allergenicity assessment of the selected dicot proteins using the FARRP20 dataset at the University of Nebraska-Lincoln^38^ revealed no identity matches of greater than 35% over 80 residues (Suppl. Table 2). The dicot ferritin query sequences were also evaluated for any eight contiguous identical amino acid matches to the allergens contained in the FARRP database. This was performed using an algorithm that generates all possible eight-word peptides from both the query and dataset proteins and evaluates each query “word” against all dataset “words” for perfect matches.

### Large-scale crop transformation and event selection using approach 1 (staining) vs approach 2 (gene copy number)

**Figure 3:**
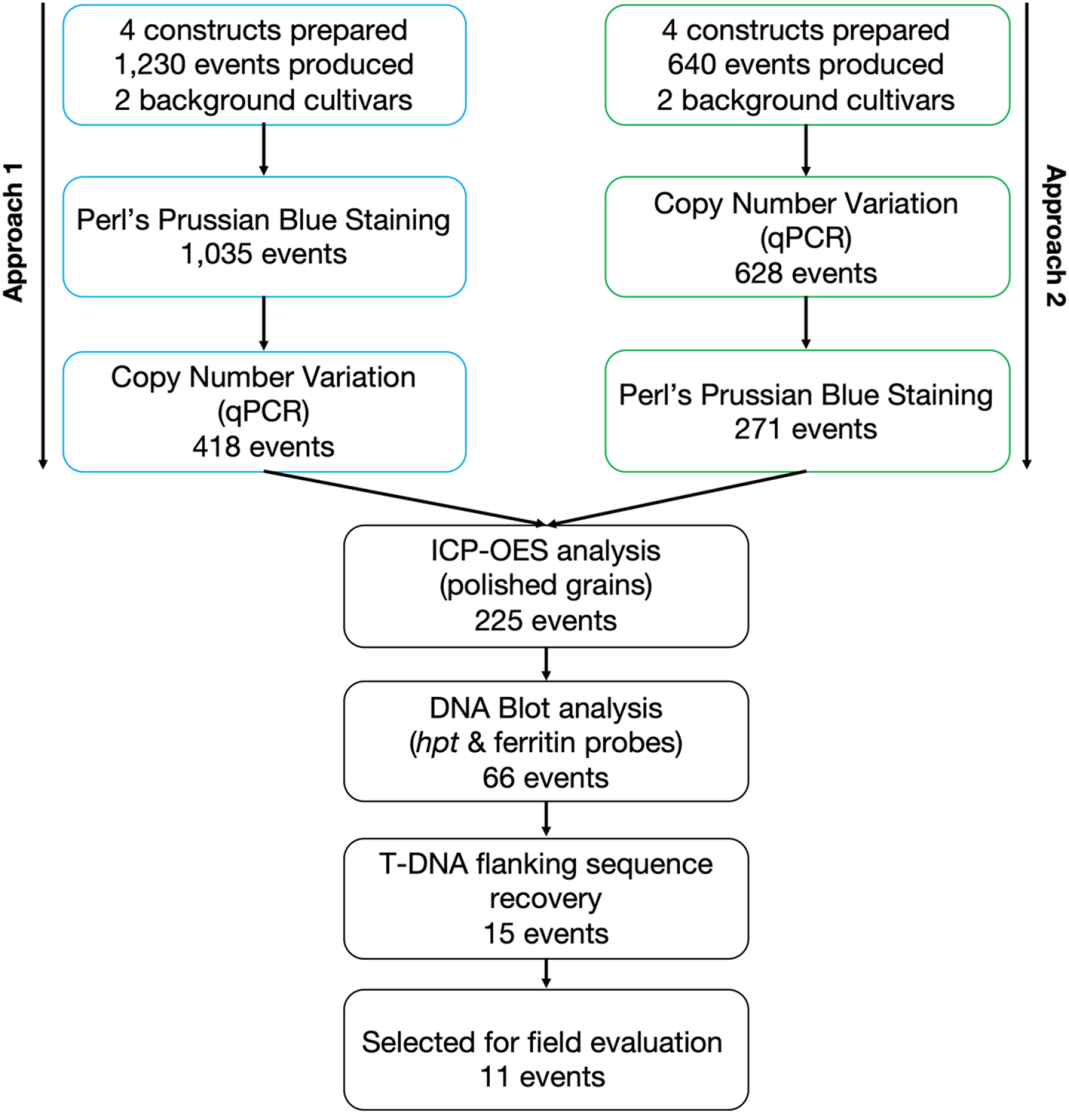
General overview of the study. In total one thousand eight hundred and seventy events were prepared from eight constructs and analyzed for high Fe and Zn in polished rice grains. Eventually eleven events that fulfilled the prerequisites for product development (high Fe and Zn content, good agronomic performance under screen house settings, simple molecular structure, and no transfer of T-DNA beyond the border sequences) were selected for further evaluation under field settings. The whole duration of the process lasted for approximately 1.5 years. *hpt*: hygromycin selectable marker; ICP-OES: inductively coupled plasma optical emission spectrometry; T-DNA: transfer DNA; qPCR: real-time polymerase chain reaction.

The scheme for the event selection can be seen in Fig. 3. Out of the 1870 transgenic events produced in total, 1035 events (or 55.3%) were analyzed using the first approach (Perl’s Prussian Blue analysis followed by copy number qPCR), whilst 628 events (or 33.6%) were analyzed using the second approach (copy number qPCR followed by Perl’ Prussian Blue staining) (Fig. 3). Also, 207 events (or 11.1%) were not considered for further analysis because they exhibited poor agronomic performance (less than 150 harvested seeds per plant) in T_0_ generation possibly due to the insertion of multiple copies of T-DNAs in the plant genome, or T-DNA position effects, or other biotic and abiotic reasons. In the first approach, initial qualitative screening for high Fe content of the polished grains resulted in the subsequent analysis of 418 events (or 42.5%) for copy number variation through qPCR (Table 1). The reduction rate following this approach fluctuated between 30 and 50% among the different constructs. In the cases of IRS1023 and IRS1030 (both in background cultivar Rc238), all the selected events from the Perl’s Prussian Blue screening were submitted for ICP analysis. Eventually, 117 low-copy events were submitted for ICP analysis (reduction rate of approximately 80% after the qPCR performance), and 18 events were selected for determination of copy numbers through DNA blot analysis using the hygromycin and the ferritin probes. In the second approach, genomic DNA was extracted from the young seedlings of 628 events, and a qPCR analysis was performed at the early vegetative stage to select low-copy events (Table 1). Eventually, 271 events were selected for subsequent Perl’s Prussian Blue analysis resulting in an overall reduction of 43.2%. The reduction rate following this approach fluctuated from approximately 40–50% amongst the respective constructs. Eventually, 108 low-copy events were submitted for ICP analysis (reduction rate of approximately 60% after Perl’s Prussian Blue screening), and subsequently, 24 events were selected for DNA blot analysis. Taking all the above into account, both approaches had similar performances facilitating the mass screening of the generated transgenic events and the subsequent selection of candidates for product development. However, the second approach that employed the performance of a qPCR for the detection of low copy number events at the seedling or early vegetative stages had a significant advantage when considering the overall cost of the screening strategy.

**Table 1:** Summary of constructs developed for transformation in cultivars Rc238 and BR28 for the purposes of the HIZR project at IRRI, as well as the actual number of the events analyzed per construct per cultivar.

				Approach 1			Approach 2						
				(already harvested grains)			(seedling stage)			DNA blot analysis			
		Background cultivar	Total events produced	Analysed by Perl's Prussian Blue	Analysed by qPCR	Reduction Rate	Analysed by qPCR	Analysed by Perl's Prussian Blue	Reduction rate	Submitted for ICP-OES	hpt probe	Ferritin probe	Selected/candidate events
IRS1023	2 ApFer + TRXNAS	Rc238	15	15	n.a	n.a	0	0	n.a	4	2	0	0
IRS1025	2 PSFer + TRXNAS	Rc238	373	339	175	0.52	0	0	n.a	31	4	0	0
		BR28	200	185	54	0.29	0	0	n.a	13	3	1	1
IRS1027	2 PVFer + TRXNAS	Rc238	395	265	131	0.49	0	0	n.a	28	3	1	1
		BR28	200	194	58	0.30	0	0	n.a	16	6	3	3
IRS1030	2 ApFer + 35SNAS	Rc238	47	37	n.a	n.a	0	0	n.a	25	24	5	5
IRS1032	2 PsFer + 35SNAS	Rc238	42	0	0	n.a	42	21	0.50	10	2	0	0
IRS1034	2 PvFer + 35SNAS	Rc238	50	0	0	n.a	50	26	0.52	21	3	0	0
IRS1134	2 PvFer + TRXintNAS	BR28	198	0	0	n.a	196	90	0.46	37	18	5	1
IRS1202	2 PsFer + TRXintNAS	BR28	350	0	0	n.a	340	134	0.39	40	1	0	0
	Total:		1870	1035	418		628	271		225	66	15	11

In total 1870 events were produced, and 1663 events were analyzed by Perl’s Prussian Blue staining and qPCR for copy number variation. Subsequently, 225 events were selected and submitted for inductively coupled plasma optical emission spectrometry to detect the Fe and Zn concentration in polished grains. The copy number of 66 samples exhibiting high Fe and Zn content was determined by DNA blot analysis using initially the hygromycin (hpt) probe and later the ferritin (transgene-of-interest) probe. Eventually, 11 events were selected for further analysis under field settings.

### Comparison of different dicot ferritin genes

**Figure 4:**
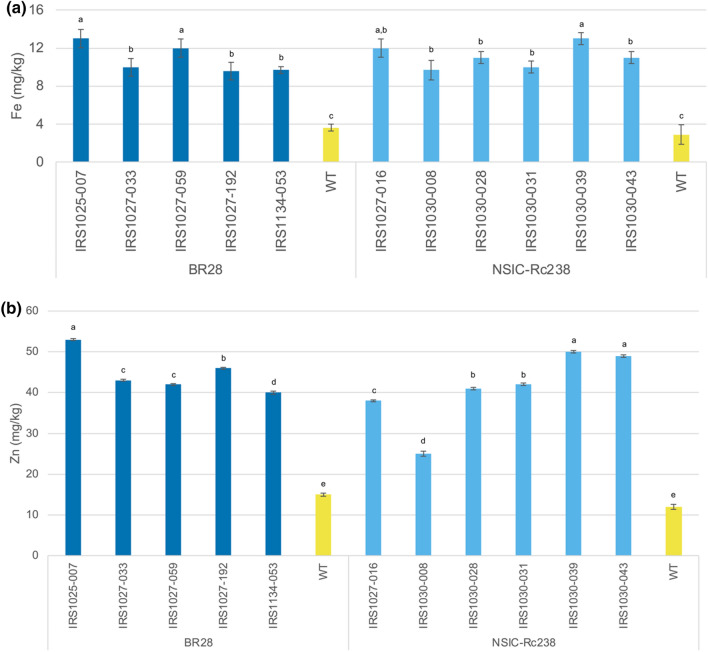
Elemental data for Fe (**a**) and Zn (**b**) were collected through inductively coupled plasma optical emission spectrometry (ICP-OES) of polished rice grains from transgenic events, grown in the screenhouse, in cv. BR28 and cv. Rc238 backgrounds, as well as their respective non-transformed controls. The values represent the mean ± standard deviation (SD) across a bulk of 150 polished grains per respective transgenic event or non-transformed control. Prior submission for elemental analysis the brown rice grains were polished through Kettmill for 55 s. The elemental analysis for all the events was conducted in Analytical Service Laboratory (ASL) at the International Rice Research Institute (IRRI) between 2015 and 2016. Means labeled with different letters differ significantly at the 5% level (Student’s T test).

In total 139 events containing either of the three dicot ferritins and the constitutive CaMV 35S promoter for *OsNAS2* (47 events for the ApFer, 42 events for the PvFer and 50 events for the PsFer) were produced in the Rc238 background cultivar. Eventually, 56 events were submitted for ICP analysis for the three different constructs. Analysis of the obtained data indicated 23 events with a concentration greater than 7 μg g^−1^ in the polished grains for the construct containing the ApFer genes (Supplementary Table 3). The highest Fe and Zn concentrations observed were 14 μg g^−1^ for event IRS103-032 and 60 μg g^−1^ for event IRS1030-024, respectively. Eventually, five events containing the *ApFer* genes fulfilled the prerequisites for advancement and further field evaluation (Fig. 4). In the case of constructs containing either the *PvFer* or *PsFer* genes, three and ten events respectively were identified with increased Fe concentration in the polished grains (Supplementary Table 3). Nevertheless, in both cases, the Fe levels remained below the threshold of 10 μg g^−1^ and hence none of these events were considered for the subsequent analysis, even if in some cases the Zn threshold level was satisfied. Taking all these into account, it is evident that ApFer expression resulted in higher concentrations of both Fe and Zn in polished rice seeds.

### Comparison of the constitutive 35S vs phloem-specific TRX promoters to the NAS gene expression

In total 783 events were produced in Rc238 background cultivar using the phloem-specific TRX promoter to drive the expression of *OsNAS2* (constructs IRS1023, IRS1025, and IRS1027). Six hundred and nineteen (619) of these events were analyzed using the first approach and eventually, 63 events were submitted for ICP analysis. Sixteen of these events (4 for constructs IRS1023 and IRS1025, and 8 for construct IRS1027), exhibited Fe concentrations higher than 7 μg g^−1^ (Supplementary Table 3). The highest Fe concentration was 12 μg g^−1^ in event IRS1027-016, whilst Zn was 43 μg g^−1^ in event IRS1023-009. Eventually, only the event IRS1027-016 was selected for further field evaluation after selection for copy number variation. Nine (9) events were selected for DNA blot analysis and 1 event (IRS1027-016) was selected for further evaluation under field conditions (Fig. 4). In the case of the constructs produced using the constitutive promoter 35S to drive expression of the *OsNAS2* gene, 139 events were produced in Rc238 background cultivar (constructs IRS1030, IRS1032, and IRS1034). Out of the 56 events that were submitted for ICP analysis, 36 events were identified with increased Fe concentrations in the polished grains (Supplementary Table 3). The highest Fe concentration (13 μg g^−1^) was observed in event IRS1030-039, whilst Zn was 60 μg g^−1^ in event IRS1030-024. Eventually, 5 events from construct IRS1030 were selected for further field evaluation (Fig. 4). Considering all the above it became evident that constitutive expression of *OsNAS2* resulted in a more efficient accumulation of Fe and Zn levels in rice polished grains.

### Comparison of the phloem-specific TRX vs TRXint promoters

We investigated the potential to increase the Fe and Zn content in polished rice grains by utilizing a phloem-specific promoter (OsTRX) in solo or in combination with an enhancer. In total 400 events were produced in the BR28 background cultivar using the phloem-specific TRX promoter to drive the expression of *OsNAS2* (constructs IRS1025 and IRS1027). Three hundred and seventy-nine (379) of these events were analyzed using the first approach and eventually, 29 events were submitted for ICP analysis. In total, 10 events were detected with Fe content greater than 7 μg g^−1^ in the polished grains (Supplementary Table 3). The highest Fe and Zn concentrations, 13 μg g^−1^ and 53 μg g^−1^ respectively, were observed in event IRS1025-007 which contained the PsFer genes (Fig. 4). For the construct containing the PvFer genes the highest Fe concentration, 12 μg g^−1^, was detected in event IRS1027-059, whereas for Zn, 48 μg g^−1^, in event IRS1027-015. Eventually, four (4) of these events fulfilled the conditions for product development and were selected for subsequent evaluation under field conditions. In a different approach, the first intron of the maize Ubiquitin gene was added immediately after the 5’ untranslated region of the OsTRX promoter to enhance its expression and subsequently the expression of *OsNAS2*. In total, 548 events were produced in BR28 background cultivar (constructs IRS1134 and IRS1202), and 536 events were analyzed using the second approach. Subsequent ICP analysis of 63 events resulted in 33 events (31 containing the PvFer and 2 with the PsFer) exhibiting high Fe and Zn levels in polished grains (Supplementary Table 3). Eventually, only one event (IRS1134-053), containing the PvFer genes and exhibiting 9.7 μg g^−1^ of Fe and 40 μg g^−1^ of Zn, was selected for further evaluation under field conditions (Fig. 4). Taking the above into consideration, we demonstrated that expression of the *OsNAS2* gene through the thioredoxin phloem-specific promoter resulted in considerably increased Fe and Zn levels in polished rice grains. Moreover, the addition of the maize Ubiquitin intron 1 at the thioredoxin promoter also enhanced the generation of events with Fe concentration greater than 7 μg g^−1^. Nonetheless, events without the enhancer (OsTRXh intron) had noticeably higher concentrations of both Fe and Zn at the polished grains. Lastly, it became evident that utilization of both PvFer and PsFer could serve as a storage mechanism for the additional Fe and hence be considered a suitable alternative for the development of high Fe and Zn rice.

### Validation of copy number analysis by DNA blot characterization of selected events

**Figure 5:**
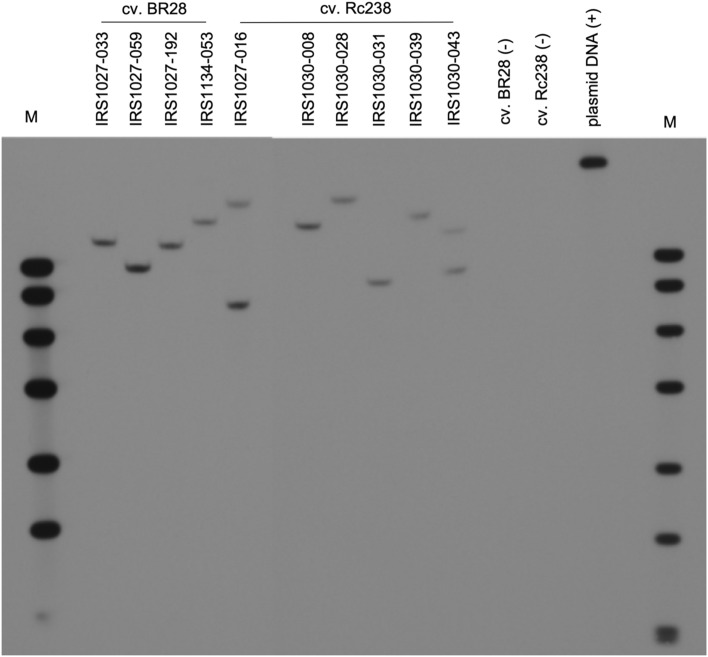
DNA blot analysis for the identification of T-DNA insertion sites in transgenic plants, using a ferritin probe. Genomic DNA from transgenic events in both cv. BR28 and cv. Rc238 backgrounds were extracted and digested with *EcoR*I enzyme prior detection with the probe. All the events have 1 copy of the T-DNA, except those events IRS1027-016 and IRS1030-043 that have 2 copies. Digested genomic DNA from non-transformed cv. BR28 and cv. Rc238 were used as negative controls. Linearized plasmid DNA of the binary vector used for the transformation was used as positive control. M: DNA Molecular Weight Marker VII, DIG labeled (Roche).

In general, a single-copy T-DNA insertion is preferred for deregulation purposes of transgenic products. To further confirm the pre-selection of low copy number events using qPCR, a DNA blot analysis was performed using a single-cutter endonuclease within the T-DNA indicated single-copy insertions for most of the selected events when the ferritin probe was used (Fig. 5; Table 2). However, two copies were observed for events IRS1027-016 and IRS1030-043. A similar result was obtained when the *hpt* was used as a probe (Table 2), for validation purposes. Event IRS1027-016 had two copies, as well as the event IRS1027-033. Whereas the event IRS1030-043 was detected to have a single-copy insertion site.

**Table 2:** DNA blot characterization overview of the selected events and simplified information for the T-DNA insertion site.

Cultivar	Event	Copy Number		
		GOI probe	*HPT* probe	Simplified T-DNA insertion site in rice genome
BR28	IRS1027-033	1	2	Promoter/5’UTR
	IRS1027-059	1	1	Non-genic region
	IRS1027-192	1	1	Promoter/5’UTR
	IRS1134-053	1	1	Promoter/5’UTR
Rc 238	IRS1027-016	2	2	Non-genic region
	IRS1030-008	1	1	Promoter/5’UTR
	IRS1030-028	1	1	Intragenic region
	IRS1030-031	1	1	Promoter/5’UTR
	IRS1030-039	1	1	Promoter/5’UTR
	IRS1030-043	2	1	Not applicable

### T-DNA insertion site

The T-DNA insertion site analysis enables the prediction of potential pleiotropic effects in the phenotype. The insertion sites of the T-DNA cassettes from ten (10) events are presented in Table 2. Analysis of the recovered flanking sequences revealed that two events, event IRS1027-059 and IRS1027-016, were in non-genic regions of Chromosomes 1 and 3, respectively. Moreover, event IRS1030-028 was in the intragenic region of the homeobox-associated leucine zipper gene, indicating possible inhibition in the expression pattern of this gene. Recovery of the T-DNA location for the remaining cassettes revealed that they integrated into the promoter and/or untranslated regions of several genes in the rice genome. Event-specific primers were designed in the harboring flanking regions and the T-DNA cassette of each specific event to amplify the expected fragments and determine their zygosity status.

### Agronomic performance and trait stability

**Figure 6:**
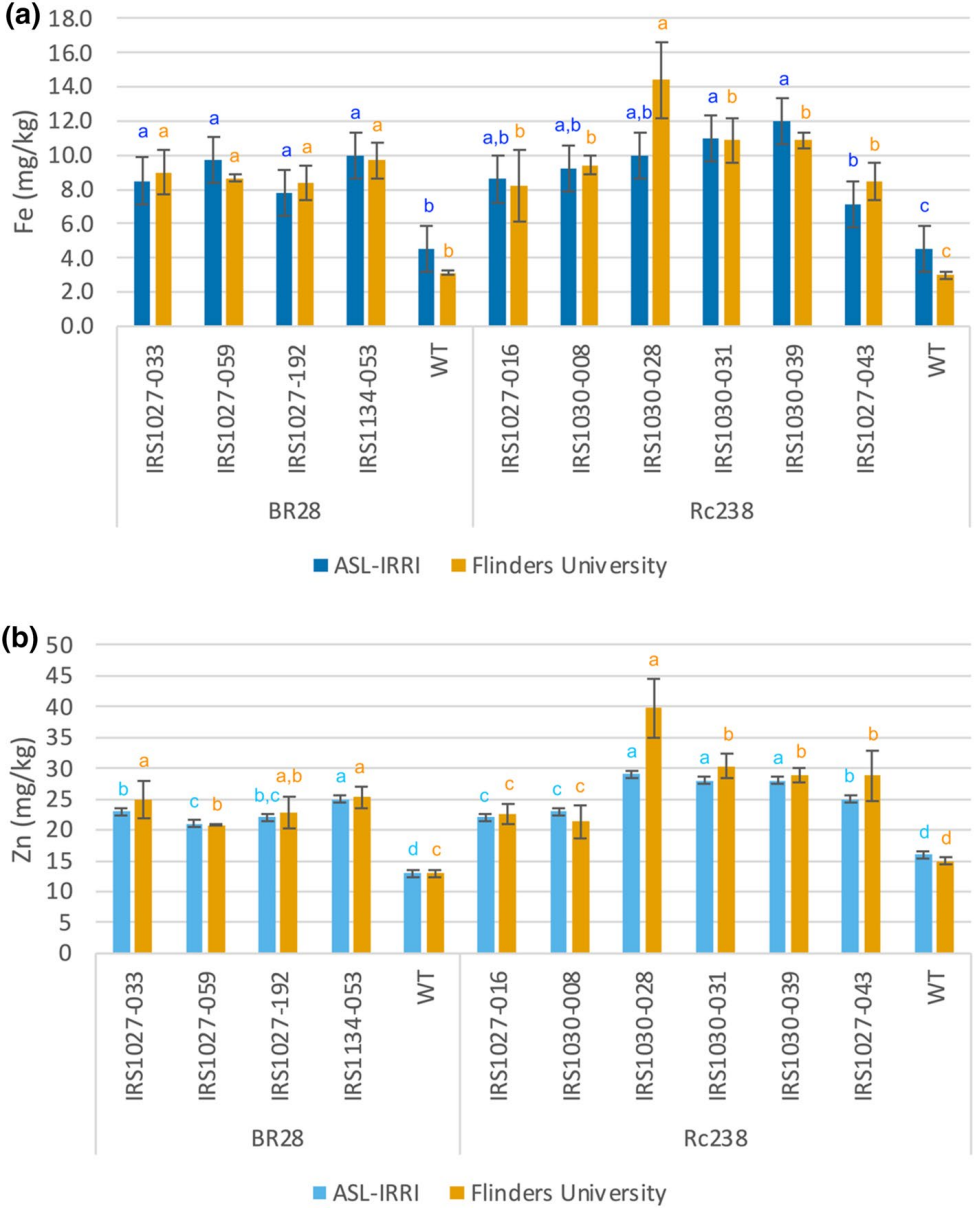
Comparison of elemental data for Fe (**a**) and Zn (**b**) were collected through inductively coupled plasma optical emission spectrometry (ICP-OES) of polished rice grains from T_2_ transgenic events in cv. BR28 and cv. Rc238 backgrounds, as well as their respective non-transformed controls, grown under field conditions at IRRI (Philippines). The values represent the mean ± standard deviation (SD) across a bulk of 150 polished grains per respective transgenic event or non-transformed control (IRRI-ASL) and the mean ± standard deviation (SD) across three replicates of a bulk of 150 polished grains per respective transgenic event or non-transformed control (Flinders University). Prior submission for elemental analysis the harvested brown rice grains were polished through Kettmill for 52 s (for events in Rc238 background) or 55 s (for events in BR28 background), and the seeds were examined visually to ensure complete removal of the aleurone layer. The elemental analysis for all the events was conducted in ASL-IRRI and the Flinders University in 2017. During the field trial, no additional Fe or Zn were supplied with the fertilizer application, despite the Zn deficient soil. Means labeled with different letters differ significantly at the 5% level (Student’ s T test).

Farmer adoption of a GM product is highly dependent on the crop agronomic performance. To assess the overall agronomic performance of the selected events, as well as the trait stability two confined tests on the field were conducted in the Philippines. Seed multiplication was completed in the paddy of a greenhouse dedicated to transgenic work. For the first field trial, approximately thirty (30) T_1_ plants per respective event were grown and their zygosity or presence/absence of the transgene-of-interest was determined by PCR using the previously listed primers. Harvested T_2_ seeds were used for preparing the materials to be assessed under field settings. Germinated T_2_ seedlings were also genotyped for zygosity and presence/absence of the transgene-of-interest (for events with elusive information regarding the T-DNA integration site) before transplanting. Elemental analysis of the polished grains harvested from 6 plants indicated that the Fe levels in polished grains ranged between 8 and 10 μg g^−1^ for events in BR28 background and between 7 and 12 μg g^−1^ for events in Rc238 background. In addition, the Zn levels in polished rice grains ranged from 21 to 27 μg g^−1^ for events in BR28 background growing in the Philippines climate and between 22 and 40 μg g^−1^ for events in Rc238 background (Fig. 6). Moreover, comparable elemental analysis that was performed at Flinders University confirmed that the Fe and Zn concentrations of polished rice grains remained at similar levels both for BR28 and Rc238 backgrounds, except for the event IRS1030-028 (Fig. 6). The maximum Fe concentration of polished rice grains at ASL-IRRI was 12 μg g^−1^ for event IRS1030-039, whereas in Flinders University it was 14 μg g^−1^ for the event in IRS1030-028, followed by 11 μg g^−1^ for events IRS1030-031 and -039, all in Rc238 backgrounds. In addition, the Zn concentration of the polished grains was 29 μg g^−1^ for events IRS1030-028 at ASL-IRRI and 40 μg g^−1^ at Flinders University. The agronomic characteristics of the previously 6 selected plants per respective event indicated that there were no statistical differences when compared to the wild-type counterpart (data not shown). Nevertheless, it is noteworthy that event IRS1025-007 had poor agronomic performance under field conditions and hence no polished grains were submitted for elemental analysis, leading to the discontinuation of this event for any future investigation (Fig. 6).

**Figure 7:**
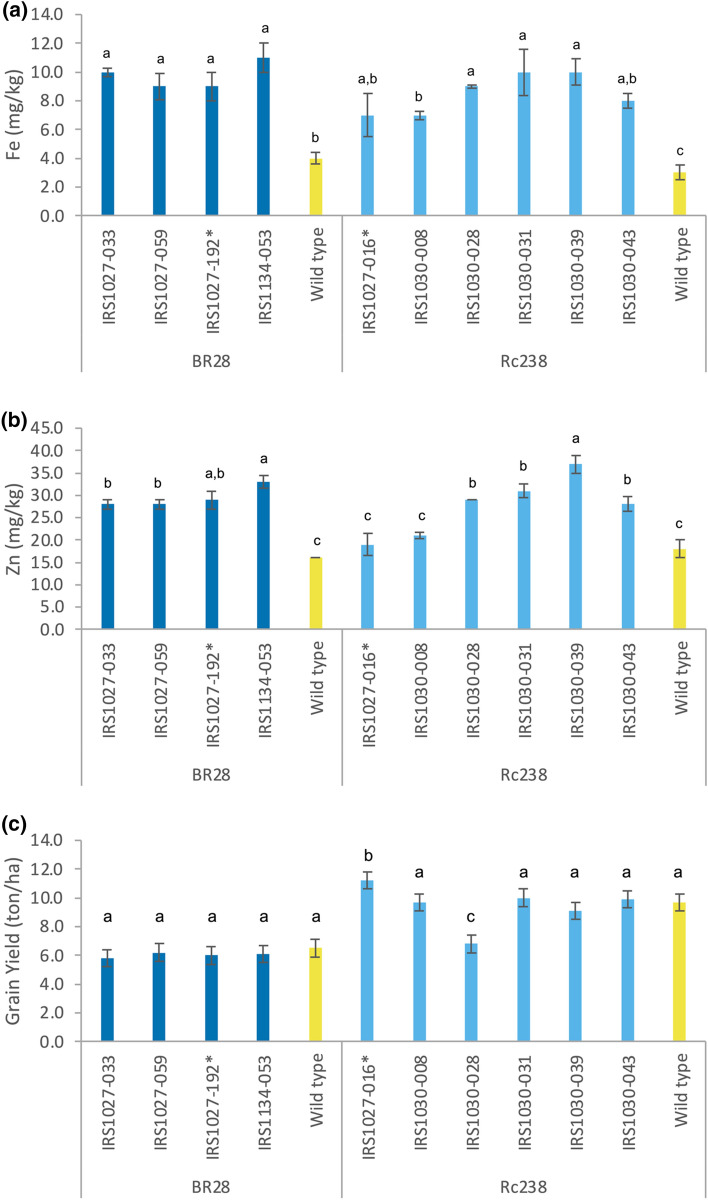
Fe (**a**) and Zn (**b**) elemental data of polished rice grains from BC_2_F_2_ transgenic events in cv. BR28 and cv. Rc238 backgrounds, as well as their respective non-transformed controls, grown under field conditions at IRRI (Philippines). Means labeled with different letters differ significantly at the 5% level (Student’s T test). Grain yield (**c**) of candidate events in cv. BR28 and Rc238 genetic background. Data were collected from a confined test on the field conducted during 2017 of BC_2_F_2_ homozygous HIZR candidate transgenic events in cv. BR28 and cv. Rc238 backgrounds and control non-transformed BR28 and Rc238, as well as the azygous controls for the respective backgrounds. Four replications were included in the field trial. The values represent the mean ± standard error (SE) across 6 different plants from each event. Data were subjected to a two-way analysis of variance (ANOVA) using entry and phenotypic parameters as factors. Means joined by the same letter group per respective background are not significantly different. Statistical significance was set at 5% level (*p* < 0.05).

For the second field trial, harvested BC_2_F_2_ seeds were used for preparing the materials to be assessed under field conditions (Supplementary Fig. 2). Germinated BC_2_F_2_ seedlings were genotyped for zygosity or presence/absence of the transgene-of-interest before transplanting, as described above. Five plants, phenotypically similar to the respective wild types, per event/line (plot) per replicate were selected for agronomic performance evaluation and elemental analysis of the polished grains (Fig. 7). Accumulation of Fe levels in polished rice grains ranged from 9 to 11 μg g^−1^ for events in BR28 background and between 7 and 10 μg g^−1^ for events in Rc238 background. Similarly, the accumulation of Zn levels in polished grains ranged from 28 to 33 μg g^−1^ for events in BR28 background, and from 19 to 37 μg g^−1^ for events in Rc238 background. Moreover, it is worth noting that no yield penalty was detected in all the events in BR28 background when compared to the wild-type counterpart (6.5 tons/ha). However, in the case of Rc238 background, the IRS1027-016 event exhibited a higher yield (11.2 tons/ha) compared to the control lines (9.7 tons/ha), whilst the IRS103-028 had a significantly lower yield (6.8 tons/ha). Taking all the above into account, we were able to demonstrate the stability of the trait in multiple generations under field settings, and agronomic performance similar to the wild-type counterparts. We finally were able to select 10 events for further advancement in the transgenic product development pipeline.

## Discussion

Consumption of polished rice as a staple food in countries with medium to a high prevalence of Fe and Zn reaches up to 150 kg/capita/year^64^. This shows the high potential of Fe- and Zn-dense rice to alleviate micronutrient Fe and Zn deficiencies, whilst complementing other current intervention strategies, in rural regions and poverty-stricken urban populations where these interventions are less effective^65^.

Our studies focused on translating the state-of-art research on Fe and Zn homeostasis genes^25–27^ to generate an impactful product by advancing on our previous studies^30^. To reduce the timeline for the advanced development stage we directly conducted a large-scale transformation in a subset of gene constructs to combine the two steps. For this purpose, eight new constructs were prepared to overexpress a set of various dicot ferritins (ApFer, PvFer, and PsFer) with high similarity to SferH-1^24^ in combination with one copy of the *OsNAS2* gene^66^. The expression of dicot ferritins was regulated by a combination of rice endosperm-specific promoters to ensure ferritin expression throughout the endosperm development process^40,51^. It is well established that translocation of Fe from flag leaves to the endosperm is highly associated with the concurrent loading of major assimilates such as sucrose^67^, and the highest rate for sucrose unloading in rice endosperm is occurring between 6 and 12 days after flowering^68^. Hence, it is conceivable to speculate that the accumulation of ferritin protein at early gain-filling stages could enable capturing and storage of the Fe unloaded during this period. Moreover, it is important to note that PvFer was utilized in earlier attempts to increase the Fe content in rice and wheat^69–73^. In rice targeted expression of *PvFer* and *AtNAS1* led to a more than sixfold increase in iron content in transgenic rice endosperm^69^, whilst in wheat, ectopic expression of *PvFer* *in solo* or in combination with *OsNAS2* resulted in the generation of several events surpassing the recommended Fe and Zn levels in wheat grains^71^. Based on the obtained results in this study, the events that were identified in cv. BR28 background and selected for further assessment predominantly expressed the PvFer and had Fe and Zn concentrations in the polished rice grains up to 12 µg g^−1^ and up to 46 µg g^−1^, respectively. Similarly, the events identified in cv. Rc238 background and selected for further assessment predominantly expressed the ApFer with Fe and Zn concentrations in the polished grains reaching up to 13 µg g^−1^ and up to 50 µg g^−1^, respectively. We were therefore able to demonstrate that we could achieve the recommended nutritional targets for Fe and Zn in polished rice grains utilizing ferritin proteins from different dicot species, other than soybean.

The *OsNAS2* gene was driven by the phloem specific OsTRXh^47^ promoter or the CaMV 35S. The *OsNAS2* gene catalyzes the synthesis of nicotianamine acid (NA) from the precursor molecule S-adenosyl methionine (SAM)^74^, whilst the NA facilitates the Fe and Zn translocation from flag leaves to the rice endosperm through the phloem^29,75^. Earlier studies have shown that constitutive overexpression of the *OsNAS2* gene resulted in a considerable increase of both Fe and Zn concentrations in rice endosperms^30,66^, but not unwanted heavy metals such as cadmium^76^. However, marginal yield penalties were observed when these events were evaluated under field settings, and this could hinder the adoption of a potential product by farmers^77^. Based on the evidence collected in this study, utilization of the OsTRXh promoter to drive the expression of *OsNAS2* led to a significant increase of Fe and Zn content in polished rice grains for events generated in cv. BR28 background, both for events containing the PvFer or the PsFer. Moreover, one candidate event (IRS1027-016) was identified for further field assessment in events generated with cv. Rc238 background. In addition, overexpression of the *OsNAS2* in combination with the ApFer resulted in the identification of five events in cv. Rc238 background, suitable for further field assessment.

To determine the accurate copy number of the introduced T-DNA in the rice genome for the respective events a DNA blot analysis was performed using a single-cutter endonuclease within the T-DNA and two probes, one on either side of the cutting site. In most cases, a single copy integration site at promoter/5’ UTR of certain genes was identified for both probes indicating a single integration of the respective T-DNAs in the genome. These observations suggest that the utilization of qPCR for copy number variation as a pre-screening strategy was useful and successful in selecting events with low or single-copy integration that will subsequently ease the deregulation procedure for the candidate events.

In addition, insertion analysis is crucial for the prediction of phenotype to select the subset of events, T-DNA insertion before the start codon could result in knockdown up to 67% of the time for protein expression in *Arabidopsis*^78^, suggesting further screening and field evaluation of these events. However, field performance will be the gold standard final validation for agronomic performance.

In the present study, we generated 1870 events in total in two broadly grown and consumed *indica* cultivars in Bangladesh (cv. BR28) and the Philippines (cv. Rc238). The initial screening of T_0_ plants was performed under screen house conditions with paddy soil to minimize the glasshouse microclimate effects on the grain Fe and Zn. The Fe and Zn concentrations of polished rice grains for events generated in cv. BR28 background was up to 13 µg g^−1^ and 53 µg g^−1^ (event IRS1025-007), respectively, approximately threefold higher compared to the wild-type counterpart. Moreover, the Fe and Zn concentration detected for events developed in cv. Rc238 background reached up to 13 µg g^−1^ and 50 µg g^−1^ (event IRS1030-039), approximately a four-fold increase compared to the wild-type counterpart. These levels of Fe and Zn in polished rice grains were consistent with our previous results for event NASFer-274^30^.

Field evaluation of the selected candidate events offers a particularly useful potential candidate gene assessment^79,80^ compared to the phenotyping performed in tightly controlled pot experiments under favorable conditions. Although the Fe levels in polished grains were maintained under field conditions (both for T_3_ and BC_2_F_3_ polished grains), our current and previous studies^81^ showed a decrease in milled grain Zn concentrations when selected events were evaluated under field settings compared to the concentrations achieved under glasshouse conditions. This is possibly due to the low Zn levels detected in our field, a condition that is often common in target countries. Nevertheless, the Zn levels of harvested polished grains from our field experiments remained consistently above the recommended nutritional targets for product development, and hence we were able to accomplish the criteria for the early development stage.

## Conclusions

We accomplished a new proof-of-concept using ferritin genes from commonly consumed dicot species (non-soybean ferritin), in combination with overexpression or phloem-specific expression of the *OsNAS2* gene, using a phase-gated approach to further advance to the early development stage on the GM product life cycle. A rigorous screening was applied to obtain a pool of 10 selected events attained the Fe and Zn nutritional targets on widely consumed elite *indica* rice cultivars (cv. BR28 for Bangladesh and cv. Rc238 for the Philippines) without yield penalty under field conditions. All the selected candidate events could potentially fulfill the 30% contribution to the EAR in the human diet for non-pregnant, non-lactating women and children 4–6 years of age and meet the requirements for the early development stage of a biotech product. Biofortified transgenic rice enriched with Fe and Zn holds enormous potential for increasing the daily intake of these important nutrients in countries where rice is consumed as a staple food. The milled grains of the selected high Fe and Zn events are colorless, similar to the addition of iodine in salt, the most successful fortification program. In addition, pyramiding it with pro-vitamin A golden rice will further multiply the impact in populations with an inadequate intake of the three elements. Currently, some of these high Fe and Zn rice events have been further advanced in the product development pipeline, and in partnership with national authorities for the respective counties, we work towards securing the permit for their commercial release. For substantial effects amongst the population, the key challenge will be to secure widespread adoption through collaboration with the health sector and the inclusion of farmer-preferred traits in the released varieties. High Fe and Zn rice has a significantly high potential to impact the amelioration of Fe deficiency anemia and Zn deficiency, and to improve the health and livelihoods of populations in rural- and in urban-poverty-stricken regions where other interventions are less effective. This is the first GM rice developed by public sector following a phase-gated approach.

## Supplementary Information

Supplementary Information 1.

Supplementary Information 2.

Supplementary Information 3.

Supplementary Information 4.

## Data Availability

All data generated or analyzed during this study are included in this published article and its supplementary information files.
