# Supplementary material for: Proof of concept and early development stage of market-oriented high iron and zinc rice expressing dicot ferritin and rice nicotianamine synthase genes
Source: Sci Rep. 2023 Jan 12;13:676. doi: 10.1038/s41598-022-26854-z (PMC9837094; doi:10.1038/s41598-022-26854-z)

**Proof of concept and early development stage of market-oriented high iron and zinc rice expressing dicot ferritin and rice nicotianamine synthase genes**

Nikolaos Tsakirpaloglou^1†^, Gela Myan Bueno-Mota^1^, Jessica Candace Soriano^1^, Erwin Arcillas^1^, Felichi Mae Arines^1§^, Su-May Yu^2^, James Stangoulis^3^, Kurniawan Rudi Trijatmiko^1^, Russell Reinke^1^, Joseph Tohme^4^, Howarth Bouis^5^, Inez H. Slamet-Loedin^1^

^1^ International Rice Research Institute (IRRI), Metro Manila, The Philippines

^2^ Institute of Molecular Biology, Academia Sinica, Naknag, Taipei, Taiwan, Republic of China

^3^ College of Science and Engineering, Flinders University, Bedford Park, SA, Australia

^4^ Bioversity International and International Center for Tropical Agriculture (CIAT) Alliance, Cali, Colombia

^5^ International Food Policy Research Institute (IFPRI) - Emeritus Fellow, Washington, DC, USA

^†^ Current address: Crop Genome Editing Laboratory (CGEL), Soil & Crop Sciences Department, Texas A&M University and Texas A&M AgriLife Research, College Station, TX, USA

^§^ Current Address: Board Institute of MIT and Harvard, Cambridge, MA, USA

Correspondence: [n.tsakirpaloglou@ag.tamu.edu](mailto:n.tsakirpaloglou@ag.tamu.edu); [i.slamet-loedin@irri.org](mailto:i.slamet-loedin@irri.org)

| 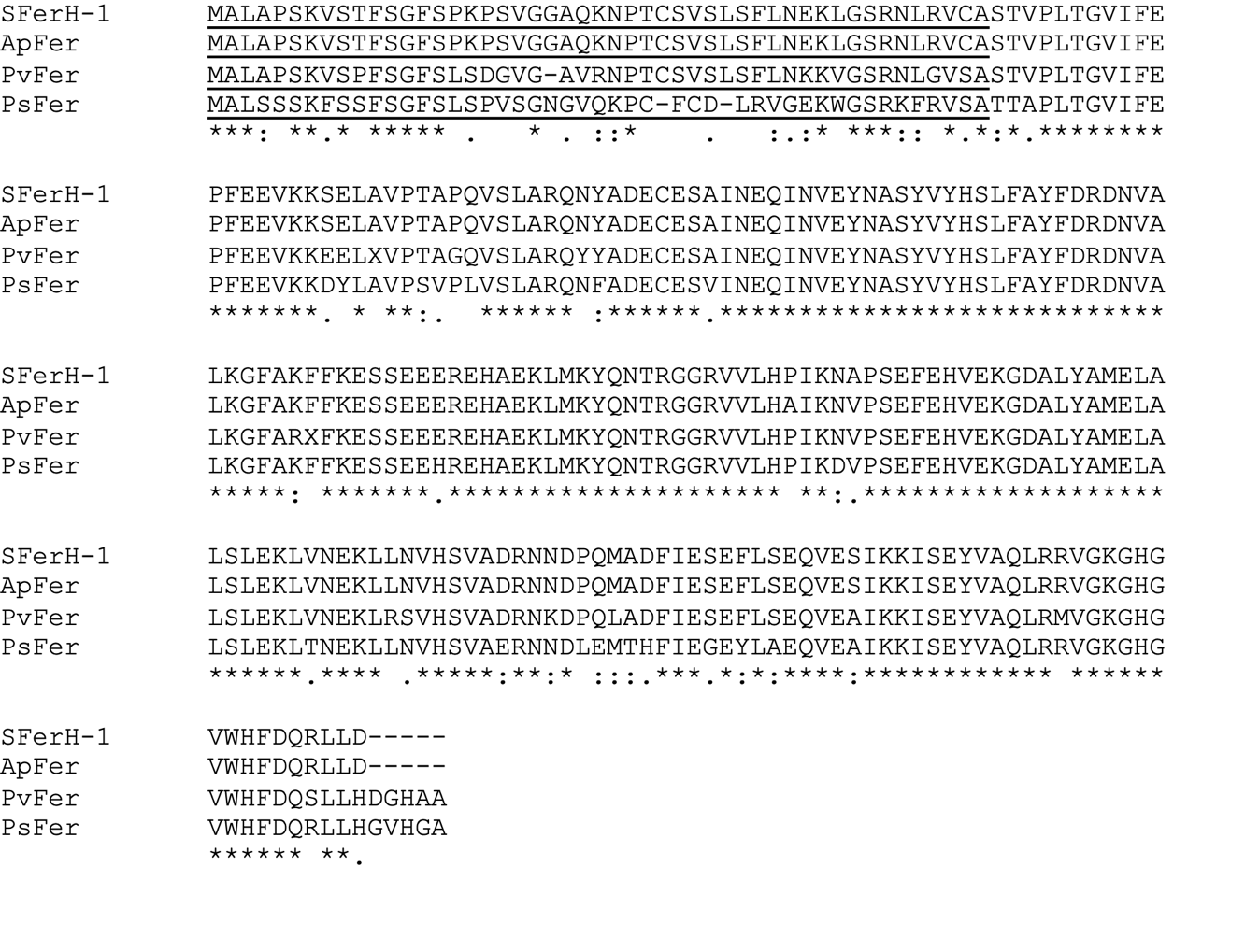 |
| --- |

**Supplementary Figure 1.** Amino acid sequence alignment of the dicot ferritins using the Clustal Omega algorithm (<https://www.ebi.ac.uk/Tools/msa/clustalo/>). Similarity of the sequences is indicated with stars (*) below the respective alignments. In all cases the transit peptide, targeting the chloroplast, is underlined.

| 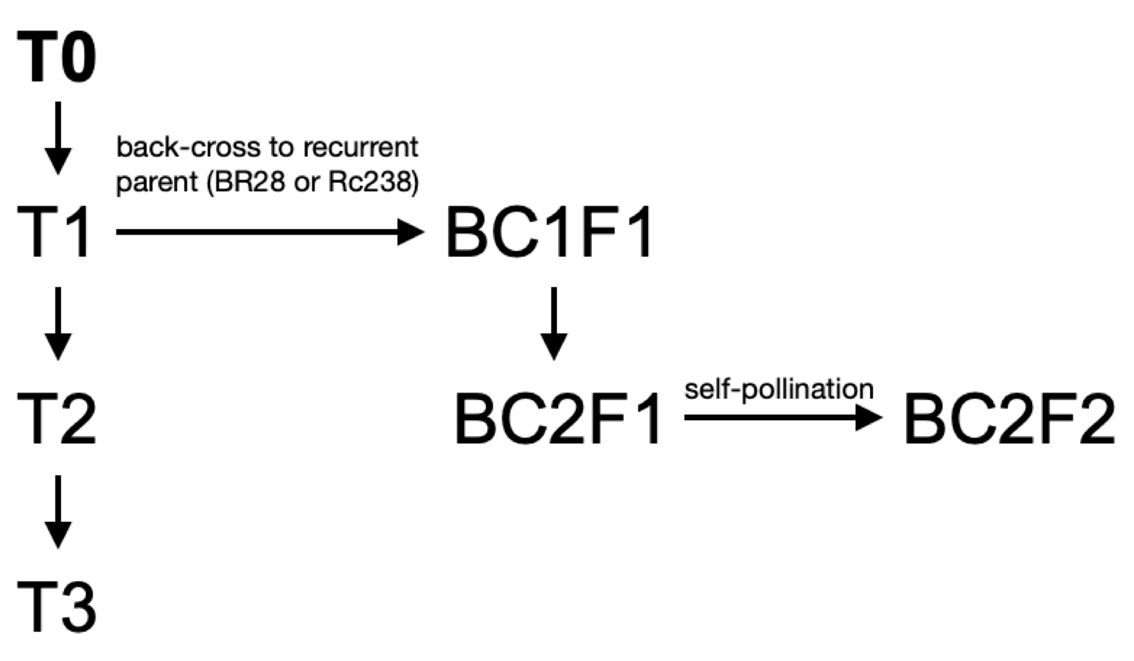 |
| --- |

**Supplementary Figure 2.** Schematic diagrams of the breeding lineage of the selected events in BR28 and Rc238 backgrounds, respectively. Generations referenced in development of the candidate HIZR varieties are shown. Materials used for field trials are T_2_ homozygous plants and BC_2_F_2_ homozygous plants.

**Supplementary Table 1.** List of dicot ferritins similar to SfeH-1. The amino acid identity is presented.

| 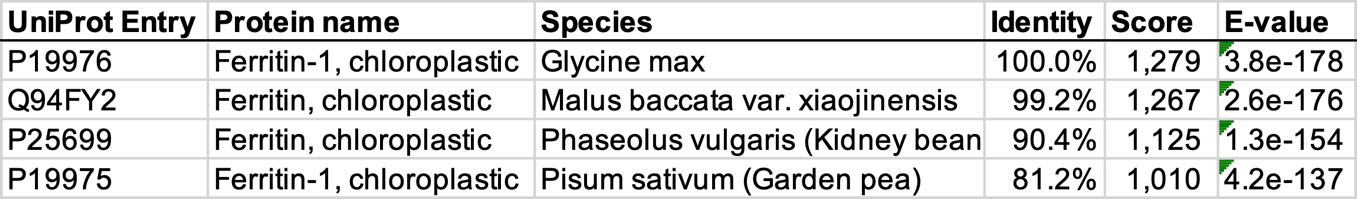 |
| --- |

**Supplementary Table 2.** Search results using the dicot ferritins query sequences against the FARRP database. The database search was performed on February 25, 2020.

| 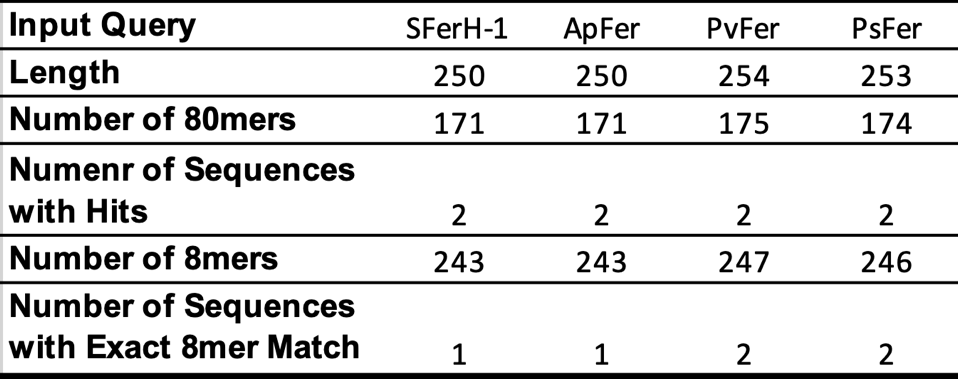 |
| --- |

**Supplementary Table 3.** Detailed summary of the events analyzed for high-Fe and -Zn content.


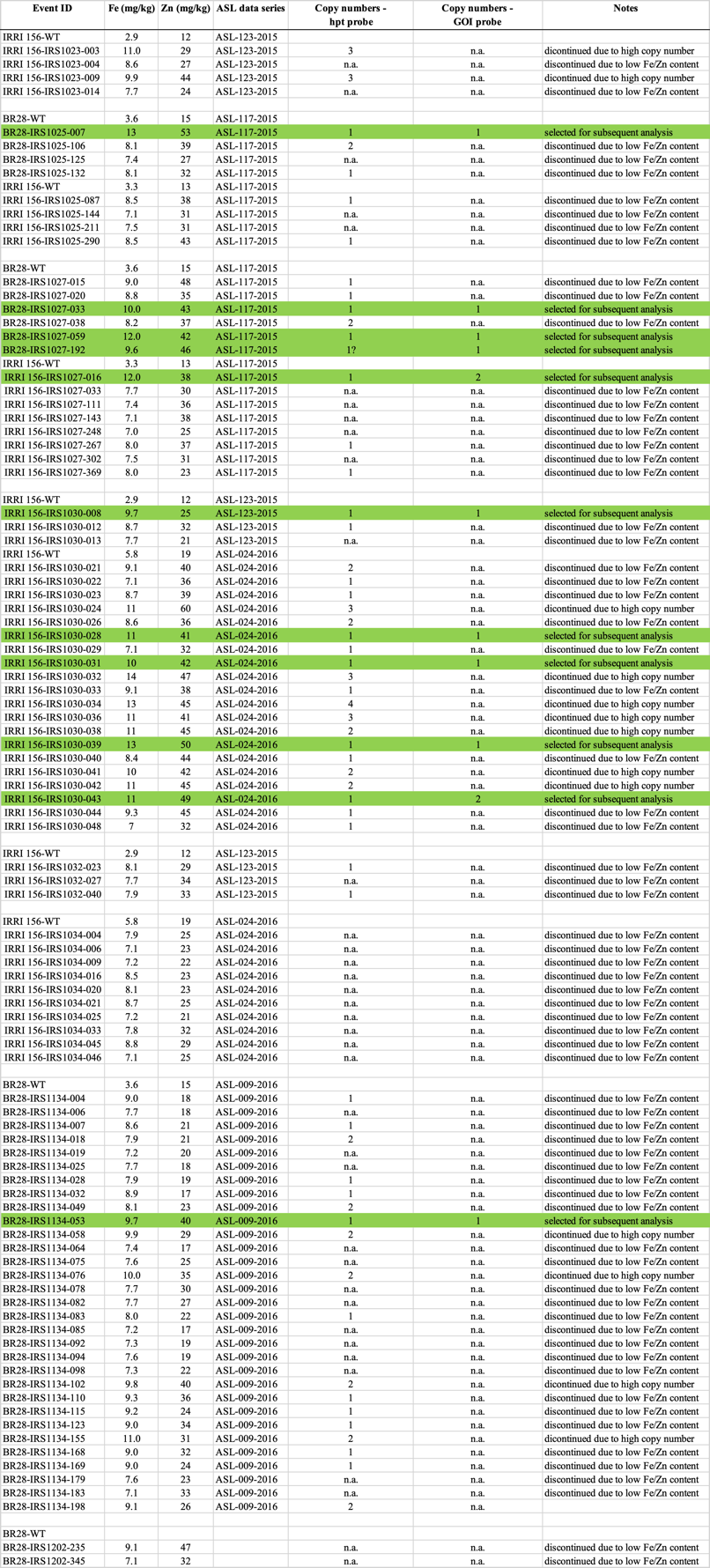

Supplement: Supplementary file 1 — Supplementary Information 1. [file 41598_2022_26854_MOESM1_ESM.docx]
